# Schistosomicidal effects of histone acetyltransferase inhibitors against *Schistosoma japonicum* juveniles and adult worms *in vitro*

**DOI:** 10.1371/journal.pntd.0012428

**Published:** 2024-08-19

**Authors:** Jing Xu, Jing-Yi Wang, Ping Huang, Zi-Hao Liu, Yu-Xin Wang, Run-Ze Zhang, Hui-Min Ma, Bi-Yue Zhou, Xiao-Yan Ni, Chun-Rong Xiong, Chao-Ming Xia

**Affiliations:** 1 Department of Parasitology, School of Basic Medical Sciences, Suzhou Medical College of Soochow University, Suzhou City, P. R. China; 2 MOE Key Laboratory of Geriatric Diseases and Immunology, Suzhou Key Laboratory of Pathogen Bioscience and Anti-infective Medicine, Suzhou Medical College, Soochow University, Suzhou City, P. R. China; 3 Jiangsu Institute of Parasitic Diseases, Wuxi City, P. R. China; Ben-Gurion University of the Negev, ISRAEL

## Abstract

**Background:**

Schistosomiasis is a relatively neglected parasitic disease that afflicts more than 250 million people worldwide, for which the control strategy relies mainly on mass treatment with the only available drug, praziquantel (PZQ). This approach is not sustainable and is a priority for developing novel drug candidates for the treatment and control of schistosomiasis.

**Methodologys/Principal findings:**

In our previous study, we found that DW-3-15, a kind of PZQ derivative, could significantly downregulate the expression of the histone acetyltransferase of *Schistosoma japonicum* (*Sj*HAT). In this study, several commercially available HAT inhibitors, A485, C646 and curcumin were screened *in vitro* to verify their antischistosomal activities against *S*. *japonicum* juveniles and adults. Parasitological studies and scanning electron microscopy were used to study the primary action characteristics of HAT inhibitors *in vitro*. Quantitative real-time PCR was employed to detect the mRNA level of *Sj*HAT after treatment with different HAT inhibitors. Our results demonstrated that curcumin was the most effective inhibitor against both juveniles and adults of *S*. *japonicum*, and its schistosomicidal effects were time- and dose dependent. However, A485 and C646 had limited antischistosomal activity. Scanning electron microscopy demonstrated that in comparison with DW-3-15, curcumin caused similar tegumental changes in male adult worms. Furthermore, both curcumin and DW-3-15 significantly decreased the *Sj*HAT mRNA level, and curcumin dose-dependently reduced the *Sj*HAT expression level in female, male and juvenile worms.

**Conclusions:**

Among the three commercially available HATs, curcumin was the most potent against schistosomes. Both curcumin and our patent compound DW-3-15 markedly downregulated the expression of *Sj*HAT, indicating that *Sj*HAT may be a potential therapeutic target for developing novel antischistosomal drug candidates.

## Author summary

Schistosomiasis is a zoonotic parasitic disease caused by *Schistosoma spp*. infection. Due to the lack of effective vaccines for schistosomiasis, PZQ is the only available drug chosen for the treatment and control of schistosomiasis in most developing countries. Drug resistance due to long-term, large-scale and single-agent use has been a great concern. Therefore, there is an urgent need for new therapies. In our previous study, we developed the patented compound DW-3-15 (patent no. ZL201110142538.2), which has significant and stable antiparasitic activity, and can significantly decrease the expression of *S*. *japonicum* histone acetyltransferase (*Sj*HAT). As we known, HATs are a class of epigenetic enzymes that are crucial for chromatin restructuring and transcriptional regulation in mammals and in parasitic development, and are thus promising targets for antischistosomal therapeutic development. In this study, we assessed three commercial HATs, including two selective p300/CBP inhibitors A485 and C646, and one natural compound, curcumin. Our results demonstrated that curcumin had the most potent worm-killing efficacy against *S*. *japonicum* juveniles and adults. In contrast, A485 and C646 displayed no effects on adult worms or mild effects on juveniles. Moreover, curcumin caused severe damage to the tegument of male worms, and the damage was similar to that of DW-3-15. Coincidentally, both curcumin and DW-3-15 significantly downregulated the expression of *Sj*HAT, indicating that *Sj*HAT might be an effective target for the development of novel therapeutic drugs. The screening and identification of selective HAT inhibitors may be a promising strategy for developing novel antischistosomal agents.

## Introduction

Schistosomiasis is a relatively neglected tropical disease caused by trematodes of the genus *Schistosoma*, mainly *S*. *mansoni*, *S*. *haematobium* and *S*. *japonicum* [1]. This disease is prevalent in Africa, the Middle East, South America, and Asia, affecting more than 250 million people worldwide and causing at least 200,000 deaths every year [1–4]. Currently, there is no vaccine available to prevent human schistosomiasis [1], and praziquantel (PZQ) is the only drug available for the treatment and control of schistosomiasis [1,3,4]. The extensive use of PZQ increases the probability of the emergence of drug resistance and worrisome data on reduced efficacy of PZQ have already been reported both in the laboratory and in the field, thus rendering the search for potential drug targets as well as novel drug candidates a strategic priority [1–4].

Schistosome has a complex life cycle and undergoes asexual proliferation and sexual reproduction. Significant morphological and metabolic changes in different developmental stages imply the existence of subtle epigenetic control of gene expression [5–7]. In addition, parasites and cancer cells have several common features, such as high metabolic and reproductive activity and the use of epigenetic processes to escape host immune surveillance [8,9]. Thus, targeting the epigenome with epidrugs developed for cancer pathologies has been considered a new promising strategy for the treatment of schistosomiasis and other parasitic diseases [9–11]. One of the most investigated posttranslational modifications is histone acetylation, where writers, readers and erasers work together to control the acetylation state of histones and consequently the transcription and expression level of different genes [12]. Histone acetyltransferases (HATs) recognized as epigenetic writers, catalyze the acetylation of histone lysine residues, which annul the positive charge of the lysine and reduce chromatin compaction, favoring transcription, whereas deacetylation, via the erasers of histone deacetylases (HDACs) has the opposite effect [13]. Relevant epigenetic targets for antischistosomal effects, such as *Sm*HDAC8 and *Sm*SIRT2, have been identified in *S*. *mansoni* [14,15]. Among them, *Sm*HDAC8 appears to be a valuable target because it is the most abundant isoform in *S*. *mansoni* and has specific and vital functions in the parasite cell cycle. Furthermore, its downregulation compromises the capacity of schistosomula to survive and become mature worms in infected mice [14,16]. However, compared with those on HDAC inhibitors, few studies on HAT inhibitors against schistosomes are currently available. Carneiro et al [17] applied a variety of technical and biological approaches to verify that histone acetylation by *Sm*CBP1 and *Sm*GCN5 creates the required epigenetic state for Smp14 transcriptional activation and eggshell formation. The HAT inhibitor PU139 prevents chromatin decondensation at the Smp14 promoter, which could further impair the production of normal *S*. *mansoni* eggs and affect the development of the female reproductive system [17]. In our previous study, we developed the patent compound DW-3-15 (patent no. ZL201110142538.2), a praziquantel (PZQ) derivative that has promising antischistosomal properties against all developmental stages of *S*. *japonicum* [18]. In the following study, we found that the *S*. *japonicum* HAT (*Sj*HAT) protein level was significantly downregulated after treatment with DW-3-15 (S1 Table and S1 Fig). In this study, we assessed the schistosomicidal efficacy of three commercially available HAT inhibitors, including A485, C646 and curcumin. A 485 is a potent, selective and drug-like p300/CBP catalytic inhibitor that selectively inhibited proliferation across lineage-specific tumor types, including several hematological malignancies and androgen receptor positive prostate cancer [19]. C646 is a widely utilized competitive p300/CBP inhibitor [19–21]. Curcumin is a natural compound that directly inhibits p300 HAT activity in a transcription-independent manner [22,23]. In addition, more than one experiment has demonstrated that curcumin has potent schistosomicidal effect on schistosomes both *in vitro* and *in vivo* [24–26].

In this study, the antischistosomal activities of the three commercially available HAT inhibitors were compared with those of DW-3-15, with the aim of verifying whether *Sj*HAT could be a potential target for developing novel antischistosomal agents.

## Materials and methods

### Ethics statement

All animal experiments were conducted in strict accordance with the recommendations in the Guide for the Care and Use of Laboratory Animals of the National Institutes of Health. All efforts were made to relieve the suffering of the experimental animals. The protocol (including mortality aspects) was approved by the Committee on the Ethics of Animal Experiments of Soochow University (Permit Number: 2007–13).

### Parasites and animals

Female ICR mice weighing 15–25 g were provided by the Experimental Animal Center of Soochow University (Suzhou, China). All mice were raised under specific pathogen-free conditions with a controlled temperature of 22°C and photoperiod (12 h light, 12 h dark). Each mouse was transcutaneously infected with 60±5 *S*. *japonicum* cercariae (a Chinese mainland strain) shedding from *Oncomelania hupensis* snails, which were provided by the Institute of Schistosomiasis Control in Jiangsu Province (Wuxi, China).

### Reagents

The praziquantel derivative DW-3-15 was synthesized by WuXi App Tec Co., Ltd. (Shanghai, China). The synthetic route of DW-3-15 was described by Wang et al. [27]. PZQ was purchased from Sigma Aldrich (St. Louis, MO, USA). A485, C646 and curcumin were purchased from Selleck Chemicals (Shanghai, China). For *in vitro* treatment, all chemicals were dissolved in dimethylsulfoxide (DMSO, Fluka, Buchs, Switzerland). Dulbecco’s modified minimum Eagle’s medium (DMEM) and penicillin/streptomycin were purchased from Life Technologies (Carlsbad, CA, USA). The newborn calf serum was obtained from Biological Industries (Cromwell, CT, USA).

### *In vitro* treatment

Worms recovered from *S*. *japonicum* cercaria infected mice at 16 days (juvenile worm) and 35 days (adult worm) by perfusion of the hepatic portal system and mesenteric veins [28], were placed in 6-well plates (Corning Costar, Corning, New York, USA) containing DMEM supplemented with 10% newborn calf serum, 100 U/ml penicillin and 100 μg/ml streptomycin, and incubated at 37°C in an atmosphere of 5% CO_2_ in air. Juvenile worms were divided into six groups, with five worms per well, each tested in triplicate, as follows: group I, untreated control, incubated with complete DMEM containing 0.1% DMSO; group II, treated with A485; group III, treated with C646; group IV, treated with curcumin; group V, treated with 100 μM DW-3-15; and group VI, treated with100 μM PZQ. Group II and group III were further subdivided into three subgroups and treated with ascending concentrations of A485 and C646 (10, 30 and 50 μM). Group IV was divided into five subgroups and treated with 20, 40, 60, 80 or 100 μM curcumin. Adult worms separated by sex received the same treatment as juveniles. All the worms were exposed to different chemicals for approximately 16h, then washed three times with pre-warmed sterile saline, and subsequently cultured in drug-free medium. At 24, 48 and 72h post-incubation, worms were observed under a dissecting microscope (SZX16, Olympus, Japan), and viability scores were assigned as described previously [29]. Briefly, the viability score of each worm ranged from 0 to 3, where a score of 3 represents that the worm moves actively and softly and has a transparent body; a score of 2 represents that the worm moves its entire body but stiffly and slowly, with the body translucent; a score of 1 represents the parasite moves partially and has an opaque appearance; a score of 0 represents that the worm was ‘dead’. For each sample, the viability score was calculated by the following formula: viability score = ∑(worm scores)/number of worms. Each experiment was repeated at least twice.

### Scanning electron microscopy

For scanning electron microscopy (SEM), worms were dehydrated in an ascending series of ethanol followed by acetone. After that, the specimens were dried, mounted on aluminum stubs, coated with gold, and then examined with a Hitachi-S4700 scanning electron microscope (Chiyodaku, Japan).

### Quantitative real-time PCR

After treatment, total RNA was extracted from cultured worms using the TRIzol RNA isolation reagent (Invitrogen, USA). The extracted total RNA was then reverse-transcribed to cDNA using the RevertAid First-Strand cDNA Synthesis Kit with Oligo (dT) 18 primers (Thermo Fisher Scientific, USA). A Real-Time PCR sequence detection system (Thermo Fisher Scientific, USA) was used for quantitative real-time PCR (qRT-PCR). The PCR products were amplified using 2×SYBR Green qRT-PCR Master Mix (Bimake, USA) with specific primers for the target genes (Table 1). The expression levels of all the transcripts were normalized to that of the housekeeping gene proteasome 26S subunit ubiquitin receptor, non-ATPase 4 (PSMD4) in the same samples.

**Table 1:** Primers for *Sj*HAT quantitative real-time PCR.

Target sequence	Forward/Reverse primer(5′→3′)
*Sj*HAT	primer-F:5′- CGACCACGTGTTAGCCAAGT-3′
	primer-R:5′- TTCCAGGCAACGTTTGCAGT-3′
PSMD4	primer-F:5′- ACTTTGAACAGGAGATGGCGA-3′
	primer-R:5′- GCCTCAGGACAACGGAACC-3′

### Statistical analysis

Statistical analysis was performed using the software package SPSS 26.0. The viability score data were expressed as the mean and standard error (mean ± SE). One-way ANOVA was used to test for differences between multiple groups, and Dunnett’s test was used to compare the differences between the two groups. A *P* value<0.05 or less was considered to indicate statistical significance.

## Results

### Schistosomicidal effect of commercial HAT inhibitors against male adult worms *in vitro*

**Fig 1:**
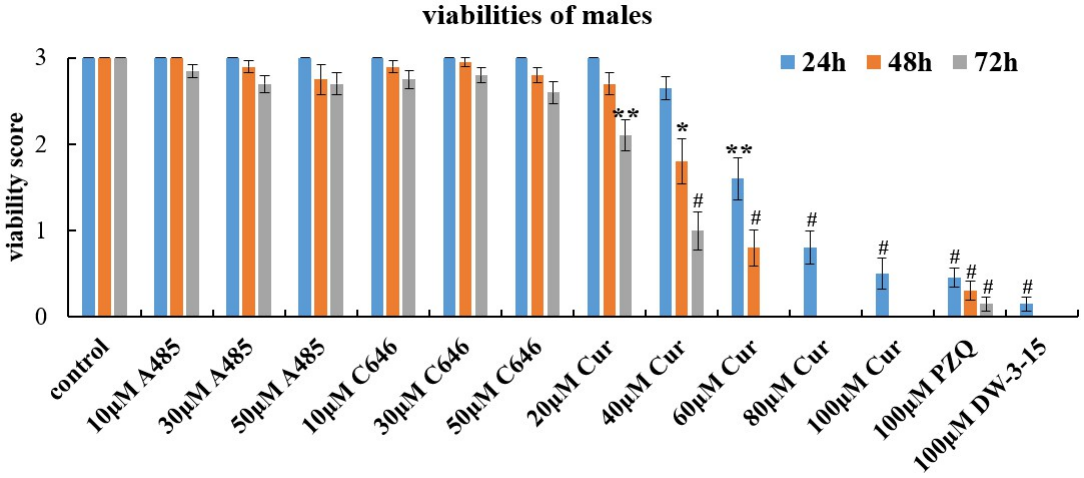
Changes in the viability of *S*. *japonicum* male worms *in vitro*. Adult male worms were exposed to different concentrations of A485, C646, curcumin, PZQ and DW-3-15 for 72h *in vitro*. The viability was evaluated using a viability score of 0–3. The control group was incubated with complete DMEM with 0.1% DMSO. The data were presented as the means ± SEs from multiple-group experiments. Statistical analysis was performed by one-way ANOVA followed by Dunnett’s test. Significant differences were indicated by *P<0.05, **P<0.005 and ^#^*P*<0.0001.

As shown in Table 2 and Fig 1, A485 and C646 had little effect on the viability of *S*. *japonicum* male adults *in vitro*. Even after treatment with 50μM A485 or 50μM C646 for 72 h, the viability reduction rates were 10% and 13.3%, respectively, and were not significantly different from that of the control group. In contrast, curcumin, demonstrated potent antischistosomal effects on male worms *in vitro*. The antischistosomal effect of curcumin was both concentration- and time dependent. With increasing curcumin concentrations (from 20 μM to 100 μM), the viability reduction rate of male decreased significantly after 24h of exposure (*F*_(13,266)_ = 105.536, *P*<0.0001). Moreover, as the incubation time increased, the male worm survival rate decreased remarkably. The antischistosomal activity of curcumin at 100 μM for 24h was similar to that of PZQ (*P* = 1.000) and DW-3-15 (*P* = 0.992). After 48h and 72h of treatment with 100 μM curcumin, all worms died, which was consistent with the effect of 100 μM DW-3-15.

**Table 2:** *In vitro* effect of different concentrations of HAT inhibitors on male adult worms of *Schistosoma japonicum*.

	Concentration (μmol/L)	24h		48h		72h	
		Worm survival rate (%)	Viability score (mean ± SE)/Viability reduction rate (%)	Worm survival (%)	Viability score (mean ± SE)/Viability reduction rate (%)	Worm survival (%)	Viability score (mean ± SE)/Viability reduction rate (%)
A485	10	100	3.00±0.00/0.0	100	3.00±0.00/0.0	100	2.85±0.08/5.0
	30	100	3.00±0.00/0.0	100	2.90±0.07/3.3	100	2.70±0.10/10.0
	50	100	3.00±0.00/0.0	100	2.75±0.10/8.3	100	2.70±0.13/10.0
C646	10	100	3.00±0.00/0.0	100	2.90±0.07/3.3	100	2.75±0.10/8.3
	30	100	3.00±0.00/0.0	100	2.95±0.05/1.7	100	2.80±0.09/6.7
	50	100	2.70±0.11/10.0	100	2.80±0.09/6.7	100	2.60±0.13/13.3
Curcumin	20	100	3.00±0.00/0.0	100	2.70±0.13/10.0	100	2.1±0.18/30.0
	40	100	2.65±0.13/11.7	80.0	1.80±0.26/40.0	55.0	1.0±0.22/66.7
	60	75.0	1.60±0.24/46.7	50.0	0.80±0.21/73.3	0.0	0.00±0.00/100
	80	60.0	0.80±0.19/73.3	0.0	0.00±0.00/100	0.0	0.00±0.00/100
	100	30.0	0.50±0.18/83.3	0.0	0.00±0.00/100	0.0	0.00±0.00/100
PZQ	100	45.0	0.45±0.11/85.0	30.0	0.30±0.11/90.0	15.0	0.15±0.08/95.0
DW-3-15	100	15.0	0.15±0.08/95.0	0.0	0.00±0.00/100	0.0	0.00±0.00/100
Control	/	100	3.00±0.00/0.0	100	3.00±0.00/0.0	100	3.00±0.00/0.0

### Schistosomicidal effect of commercial HAT inhibitors against female adult worms *in vitro*

**Fig 2:**
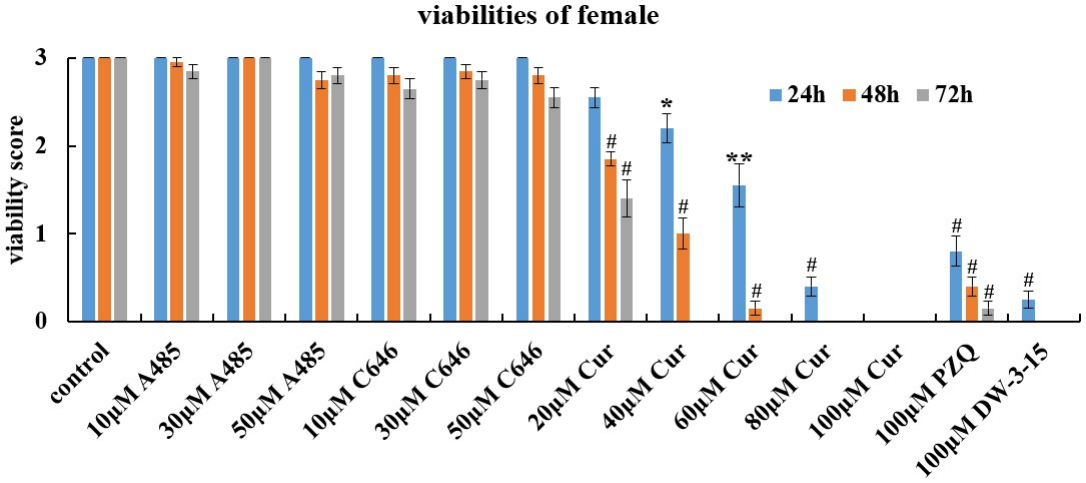
Changes in the viability of *S*. *japonicum* female worms *in vitro*. Adult female worms were exposed to different concentrations of A485, C646, curcumin, PZQ and DW-3-15 for 72h *in vitro*. The viability was evaluated using a viability score of 0–3. The control group was incubated with complete DMEM with 0.1% DMSO. The data were presented as the means ± SEs from multiple-group experiments. Statistical analysis was performed by one-way ANOVA followed by Dunnett’s test. Significant differences were indicated by *P<0.05, **P<0.001 and ^#^*P*<0.0001.

Among the three commercial HAT inhibitors, A485 and C646 demonstrated very little activity against female worms of *S*. *japonicum*, and the viability reduction rate did not exceed 15.0% after treatment with different doses of A485 and C646 for 72h. However, curcumin, could exert potent schistosomicidal effect on female worms in a dose- and time dependent manner. After 24 h of exposure, all the female worms died at the concentration of 100 μM curcumin. With the incubation period extended to 72h, the lethal dose of curcumin decreased to 40 μM. The schistosomicidal activity of curcumin against females was similar to that of 100 μM DW-3-15, but slightly greater than that of 100 μM PZQ (Table 3 and Fig 2).

**Table 3:** *In vitro* effect of different concentrations of HAT inhibitors on female adult worms of *Schistosoma japonicum*.

	Concentration (μmol/L)	24h		48h		72h	
		Worm survival rate (%)	Viability score (mean ± SE)/Viability reduction rate (%)	Worm survival (%)	Viability score (mean ± SE)/Viability reduction rate (%)	Worm survival (%)	Viability score (mean ± SE)/Viability reduction rate (%)
A485	10	100	3.00±0.00/0.0	100	2.95±0.05/1.7	100	2.85±0.08/5.0
	30	100	3.00±0.00/0.0	100	3.00±0.00/0.0	100	3.00±0.00/0.0
	50	100	3.00±0.00/0.0	100	2.75±0.10/8.3	100	2.80±0.09/6.7
C646	10	100	3.00±0.00/0.0	100	2.80±0.09/6.7	100	2.65±0.11/11.7
	30	100	3.00±0.00/0.0	100	2.85±0.08/5.0	100	2.75±0.10/8.3
	50	100	3.00±0.00/0.0	100	2.80±0.09/6.7	100	2.55±0.11/15.0
Curcumin	20	100	2.55±0.11/15.0	95.0	1.85±0.18/38.3	80	1.4±0.21/53.3
	40	100	2.20±0.17/26.7	70.0	1.00±0.18/66.7	0.0	0.00±0.00/100
	60	75.0	1.55±0.25/48.3	15.0	0.15±0.08/95.0	0.0	0.00±0.00/100
	80	40.0	0.40±0.11/86.7	0.0	0.00±0.00/100	0.0	0.00±0.00/100
	100	0.0	0.00±0.00/100	0.0	0.00±0.00/100	0.0	0.00±0.00/100
PZQ	100	60.0	0.80±0.17/73.3	40.0	0.40±0.11/86.7	15.0	0.15±0.08/95.0
DW-3-15	100	25.0	0.25±0.10/91.7	0.0	0.00±0.00/100	0.0	0.00±0.00/100
Control	/	100	3.00±0.00/0.0	100	3.00±0.00/0.0	100	3.00±0.00/0.0

### Schistosomicidal effect of commercial HAT inhibitors against juvenile worms *in vitro*

**Fig 3:**
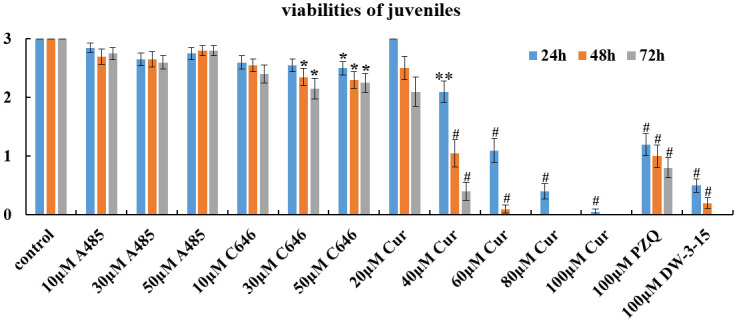
Changes in the viability of *S*. *japonicum* juveniles *in vitro*. Juveniles were exposed to different concentrations of A485, C646, curcumin, PZQ and DW-3-15 for 72h *in vitro*. The viability was evaluated using a viability score of 0–3. The control group was incubated with complete DMEM with 0.1% DMSO. The data were presented as the means ± SEs from multiple-group experiments. Statistical analysis was performed by one-way ANOVA followed by Dunnett’s test. Significant differences were indicated by *P<0.05, **P<0.005 and ^#^*P*<0.0001.

As shown in Table 4 and Fig 3, A485 had light effects on juvenile worms *in vitro*. After treatment with different concentrations of A485 for 24h, 48h and 72h, the reduction in viability of the juvenile worms was relatively low, ranging from 5.0% to 13.3%. C646 had light schistosomicidal activity against schistosomula. The viability reduction rate of juvenile worms ranged from 13.3% to 28.3%. However, all the worms survived after incubation with different concentrations of A485 and C646. Unlike in A485 and C646, curcumin had a prominent worm-killing effect on *S*. *japonicum* juveniles *in vitro*. With increasing concentrations of curcumin, the worm survival rate decreased significantly, and as the cultivation period increased to 72h, all the juveniles died at a concentration of 60 μM.

**Table 4:** *In vitro* effect of different concentrations of HAT inhibitors at on juveniles of *Schistosoma japonicum*.

	Concentration (μmol/L)	24h		48h		72h	
		Worm survival rate (%)	Viability score (mean ± SE)/Viability reduction rate (%)	Worm survival (%)	Viability score (mean ± SE)/Viability reduction rate (%)	Worm survival (%)	Viability score (mean ± SE)/Viability reduction rate (%)
A485	10	100	2.85±0.08/5.0	100	2.70±0.13/10.0	100	2.75±0.10/8.3
	30	100	2.65±0.11/11.7	100	2.65±0.13/11.7	100	2.60±0.11/13.3
	50	100	2.75±0.10/8.3	100	2.80±0.09/6.7	100	2.80±0.09/6.7
						100	
C646	10	100	2.60±0.11/13.3	100	2.55±0.11/15.0	100	2.40±0.15/20.0
	30	100	2.55±0.11/15.0	100	2.35±0.15/21.7	100	2.15±0.18/28.3
	50	100	2.50±0.11/16.7	100	2.30±0.14/23.3	100	2.25±0.16/25.0
Curcumin	20	100	3.00±0.00/0.0	95.0	2.50±0.20/16.7	85.0	2.1±0.25/30.0
	40	100	2.10±0.18/30.0	60.0	1.05±0.23/65.0	30.0	0.40±0.15/86.7
	60	75.0	1.10±0.20/63.3	10.0	0.10±0.07/96.7	0.0	0.00±0.00/100
	80	35.0	0.40±0.13/86.7	0.0	0.00±0.00/100	0.0	0.00±0.00/100
	100	5.0	0.05±0.05/98.3	0.0	0.00±0.00/100	0.0	0.00±0.00/100
PZQ	100	75.0	1.2±0.19/60.0	65.0	1.0±0.19/66.7	60.0	0.8±0.17/73.3
DW-3-15	100	50.0	0.50±0.11/83.3	20.0	0.20±0.09/93.3	0.0	0.00±0.00/100
Control	/	100	3.00±0.00/0.0	100	3.00±0.00/0.0	100	3.00±0.00/0.0

The results showed that among the three commercial HAT inhibitors, curcumin had the most significant worm-killing effect on juveniles, male and female adult worms of *S*. *japonicum in vitro*. The schistosomicidal effect of curcumin was both dose- and time dependent.

### Morphological change analysis by SEM

**Fig 4:**
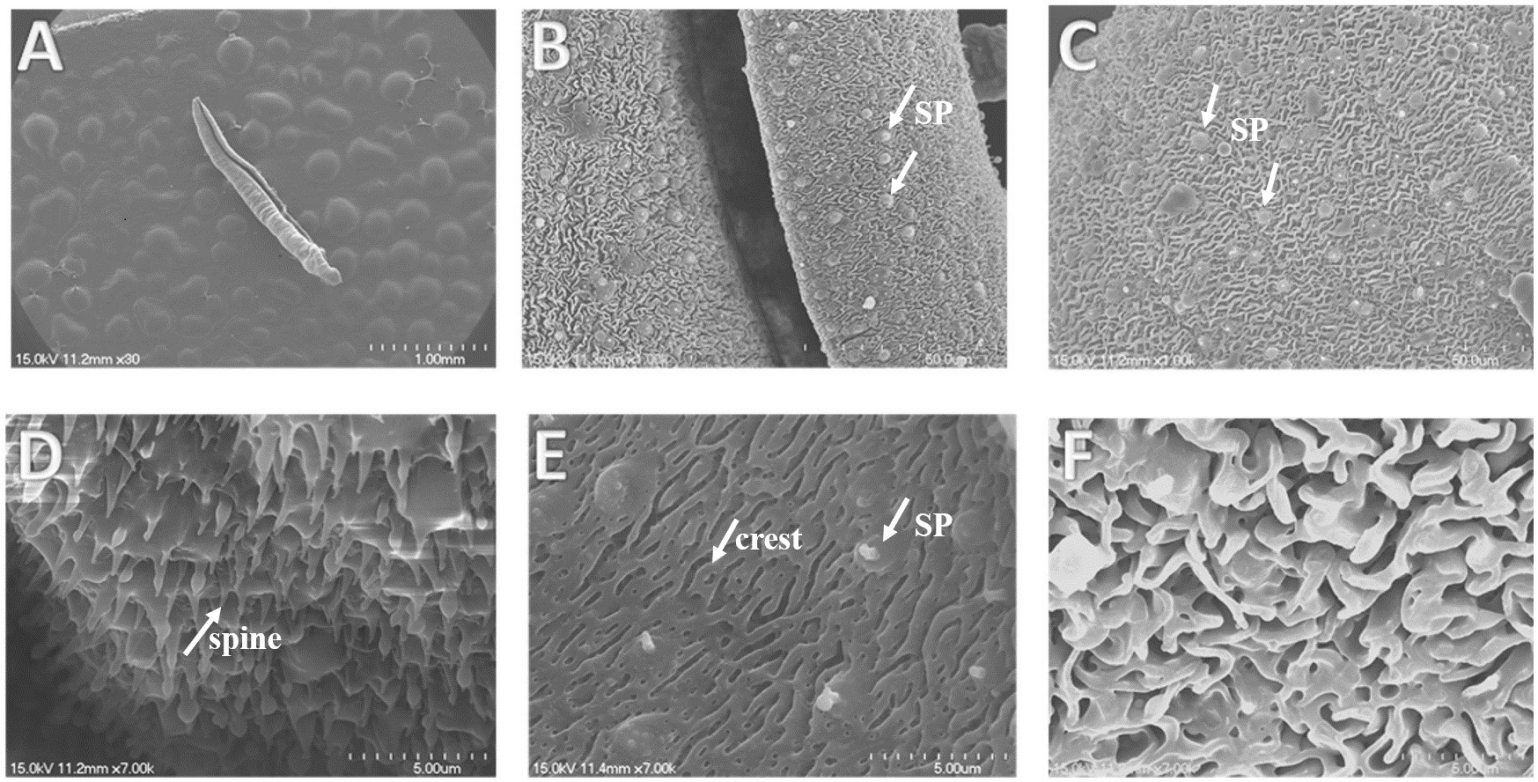
Scanning electron micrographs of the tegument of *S*. *japonicum* males in the control group. (A) Gentle panorama of a male worm in the control group after incubation in complete DMEM for 72h; (B) normal morphology of the gynecophoral canal of the male worm: the tegument is intact, and the crests with sensory papillae (SPs) are uniform along the body; (C) integrated outer wall of the gynecophoral canal: the crests and papillae (SPs) are typical; (D) ventral sucker: the spines are uniformly arranged; (E)-(F) numerous tegumental crests with sensory papillae (SPs) distributed orderly along the body surface. Scale bars: A: 1 mm; B: 50 μm; C: 50 μm; D: 5 μm; E: 5 μm; F: 5 μm.

**Fig 5:**
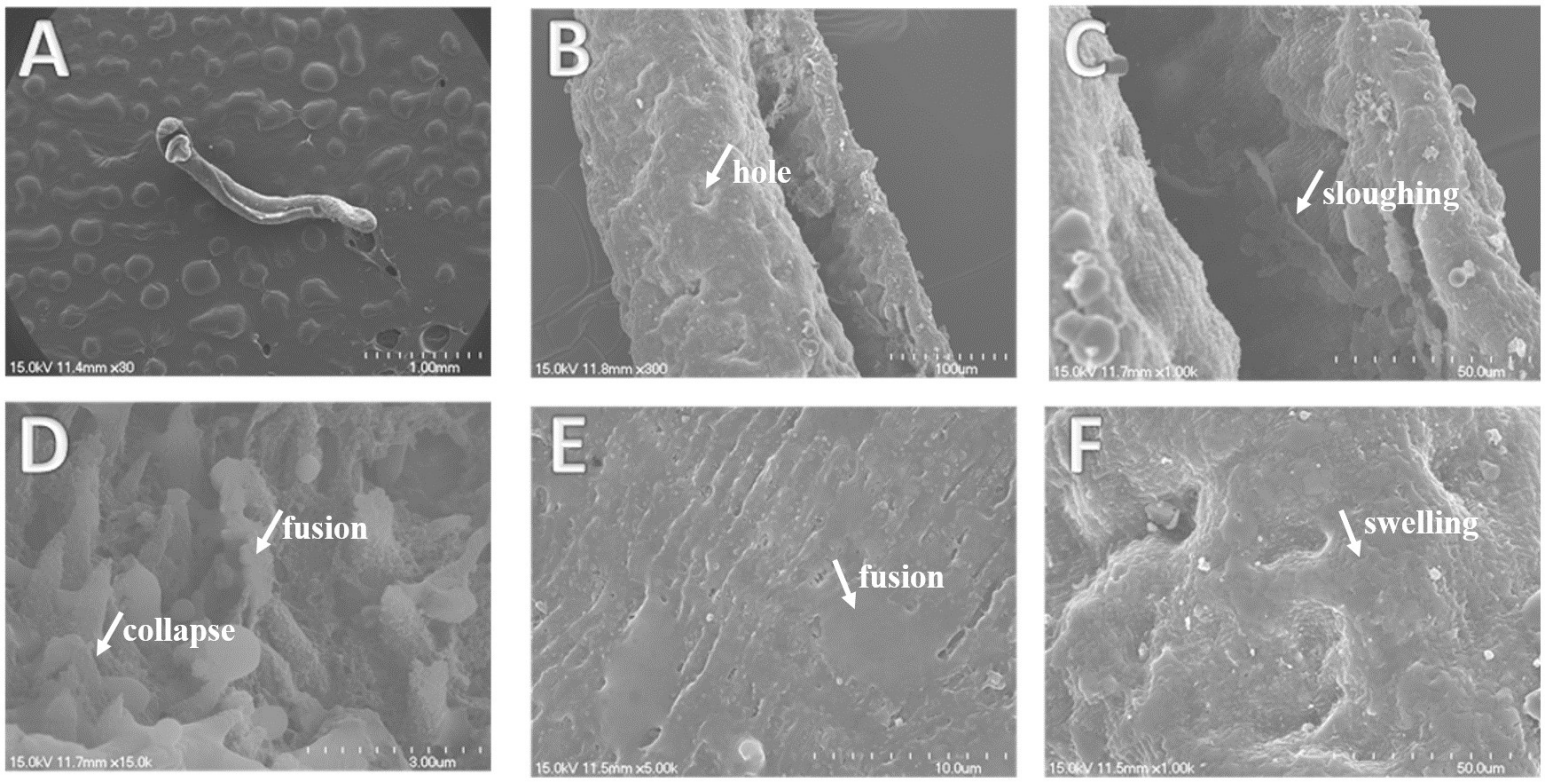
Scanning electron micrographs of the tegument of male *S*. *japonicum* worms exposed to 100 μM curcumin for 72h. (A) The whole worm was swollen and shrunken; (B) hole-shaped erosions appear on the outside wall of the gynecophoral canal; (C) extensive sloughing (SL) is observed on the inner wall of the gynecophoral canal; (D) disarrangement, fusion and collapse of the spines in the ventral sucker are obvious; (E)-(F) swelling and fusion of the crests on the mid-body tegument are prominent. Scale bars: A: 1 mm; B: 100 μm; C: 50 μm; D: 3 μm; E: 10 μm; F: 50 μm.

Under SEM, male worms of *S*. *japonicum* from the control group showed normal ultrastructural features (Fig 4). The tegument of the worm was intact, and the crests with sensory papillae were uniformly arranged along the body (Fig 4B, 4C, 4D, 4E and 4F). Apically directed spines with sensory papillae were distributed uniformly in the ventral sucker (Fig 4D). After treatment with 100 μM curcumin, the whole male worm swelled and shrank (Fig 5A), and the gynecophoral canal was severely injured with obvious swelling. Many hole-shaped erosions appeared on the outer wall of the gynecophoral canal (Fig 5B), and extensive sloughing was observed on the inner wall of the gynecophoral canal (Fig 5C). The spines within the ventral sucker were disformed, collapsed or fused (Fig 5D). Instead of uniformly arranged crests, severe edema and fusion of the crests in the tegument of the mid-body were prominent (Fig 5E and 5F).

### Curcumin significantly reduced *Sj*HAT expression in males, females and juveniles *in vitro*

**Fig 6:**
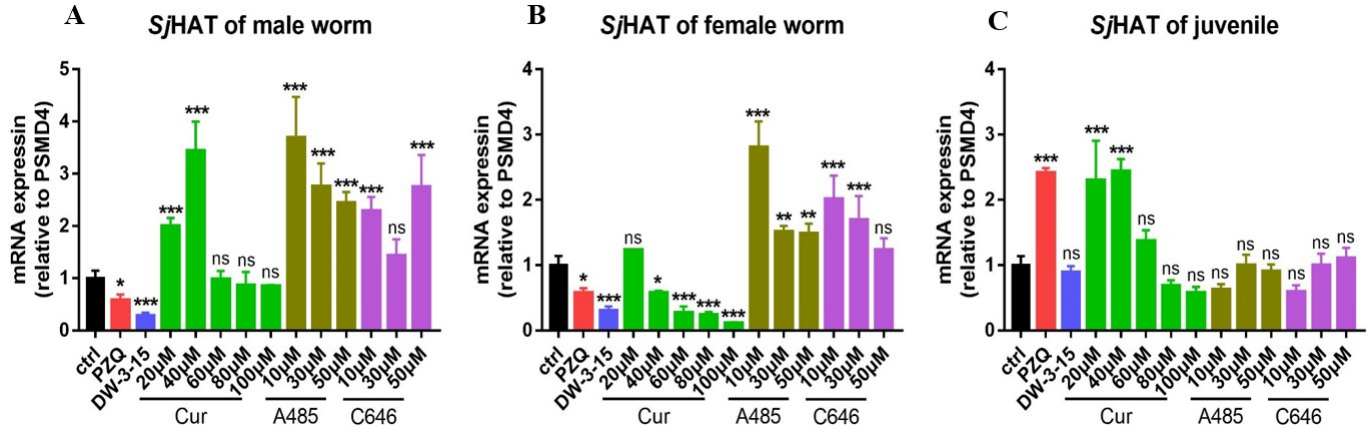
Transcriptional changes in *Sj*HAT after treatment with different concentrations of A485, C646 and curcumin in males (A), females (B) and juveniles (C) of *S*. *japonicum* detected by real-time quantitative PCR. The control group was incubated with complete DMEM with 0.1% DMSO. ‘Cur’ represents the curcumin treatment group. *represents a significant difference compared to the control group, *P*<0.05. **represents a significant difference compared to the control group, *P*<0.005. ***represents a significant difference compared to the control group, *P*<0.0001. ‘ns’ represents no difference compared with the control group.

As shown in Fig 6A, the expression level of *Sj*HAT in male worms after 72 h of exposure to 20 μM and 40 μM curcumin was significantly higher than control group (*P*<0.0001). With increasing concentrations of curcumin, the expression level of *Sj*HAT decresed, but no significant difference was observed compared with that in the control group (Fig 6A). In female worms, the expression level of *Sj*HAT decreased with increasing concentrations of curcumin. At a concentration of 40 μM, the transcription level of *Sj*HAT was significantly lower than that in the control group (*P<*0.05, Fig 6B). As the concentration of curcumin increased to 100 μM, the expression level of *Sj*HAT decreased compared with that in the 100 μM DW-3-15 treated group and was significantly different from that in the control group (*P<*0.0001, Fig 6B). In contrast, after incubation with different concentrations of A485 and C646, the expression level of *Sj*HAT in males and females was higher than that of control group (Fig 6B). While for juveniles, both DW-3-15 and curcumin at concentration of 100 μM caused little change in the transcription of *Sj*HAT compared with that in the control group (Fig 6C). After treatment with different concentrations of A485 and C646, there was no significant difference compared with the control group (Fig 6C).

## Discussion

*Schistosoma* species have a complex life cycle, and their development is regulated by epigenetic processes [30]. Therefore, enzymes involved in epigenetic modifications are viable drug targets. Many researchers have focused on epigenetic targets for schistosome drug discovery with most attention directed towards the identification and inhibition of histone modifying enzymes (HMEs) [5,6,31,32]. It is well known that histone acetylation is regulated by histone acetyltransferases (HATs) and histone deacetylase (HDACs). Furthermore, the identification of HDACs and HATs in schistosomes [15,33,34] has revealed their potential as therapeutic targets. In our previous study, we have found that DW-3-15, a kind of PZQ derivative, could significantly downregulate the expression of *Sj*HAT (S1 Table and S1 Fig). *Sj*HAT belongs to the HAT1 family, which is one of the earliest discovered HATs that primarily acetylates lysine 5 and lysine 12 of histone H4 (H4K5 and H4K12), and is involved in replication-dependent chromatin assembly [35]. In this study, the antischistosomal activity of three commercially available HAT inhibitors, A485, C646 and curcumin was assessed, with the aim of verifying the feasibility of using *Sj*HAT as a novel therapeutic target for schistosomiasis. All the three mentioned compounds were reported to be highly selective inhibitors of p300/CBP [19–23]. Our *in vitro* results demonstrated that both A485 and C646 had little antischistosomal activity against *S*. *japonicum* adult worms (Figs 1 and 2, Tables 2 and 3). While for juveniles, A485 also had a limited effect, but C646 at a concentration of 50 μM slightly reduced the viability compared with that of the control group after 72h of exposure (Fig 3 and Table 4). Although several reports have shown that A485 and C646 have promising anticancer effects *in vitro* [19,20], they do not retain the same activity against multicellular organisms, such as *Schistosoma* species. This phenomenon was confirmed by Wang et al [36]. They examined the activity of 10 μM A485 on *S*. *mansoni* adult worms cultured *in vitro* and found no effect on worm movement or attachment [36]. By contrast, curcumin, a natural compound with an inhibitory effect on histone acetylation [22,23], demonstrated the most potent schistosomicidal effect against both adult worms and juveniles. The antischistosomal activity of curcumin was both dose- and time-dependent, and it caused the death of all worms at a concentration of 60 μM after 72h of exposure (Figs 1–3, Tables 2–4). Our results were consistent with the previous reports that using curcumin as a schistosomicidal agent against schistosomes [24–26]. It is intriguing to note that except for curcumin, only DW-3-15 at 100 μM can cause 100% death of schistosomula and adult worms (Tables 2–4).

In addition to the similar biological effects of curcumin and DW-3-15, the ultrastructural changes in the tegument of male adult worms treated with 100 μM curcumin were also similar to those in DW-3-15. Our SEM observation revealed that curcumin caused severe damage to the tegument of male adult worms, including sloughing of the tegument on the inner wall of the gynecophoral canal, swelling and fusion of the spines and crests on the mid-body tegument (Fig 5C–5F). All these changes can also be observed in worms treated with 100 μM DW-3-15 [27,37], indicating that DW-3-15 and curcumin might share some common antischistosomal mechanism.

The mRNA level of *Sj*HAT after 72h of exposure to 100 μM DW-3-15 and 100 μM curcumin further confirmed this hypothesis. Both DW-3-15 and curcumin significantly inhibited the expression of *Sj*HAT in female worms (Fig 6B). Although no significant difference of *Sj*HAT level was observed in male worms, the *Sj*HAT mRNA level was lower than that of control group (Fig 6A). While for juveniles, both DW-3-15 and curcumin had little effect on *Sj*HAT mRNA level in comparison with control group (Fig 6C). In contrast, the *Sj*HAT level in adult worms treated by different concentrations of A485 and C646 was significantly higher than those in the control group (Fig 6A and 6B). The opposite expression pattern of *Sj*HAT mirrors their antischistosomal activity. Among the compounds, curcumin and DW-3-15 are the most potent schistosomicidal agents against both male and female adults (Tables 2 and 3), even better than the effective drug PZQ. Accordingly, the mRNA level of *Sj*HAT in worms treated by 100 μM DW-3-15 and curcumin was lower than that of negative control group. Conversely, both A485 and C646 had little effect against adult worms (Tables 2 and 3), their *Sj*HAT level was significantly higher than those in the control group (Fig 6A and 6B). Although A485 and C646 had promising anti-cancer activity *in vitro*, they could not inhibit the expression of *Sj*HAT. This might be partially attributed to their selective inhibition of p300/CBP and their weaker effect on inhibiting other types of HATs, such as HAT1. As a natural compound, curcumin may not selectively act on a single target, such as A485 and C646, but may have multiple targets. In addition to inhibiting the activity of P300/CBP, curcumin can also ameliorate PRMT5-MEP50 arginine methyltransferase expression by decreasing the expression of Sp1 and NF-YA transcription factors in the A549 and MCF-7 cells [38]. Furthermore, it has been reported that curcumin can inhibit HDAC activity and downregulate the expression of HDAC types 1, 2, 3, 4, 5, 6, 8 and 11 in different cancer cell lines and mice [39]. Our results demonstrate that curcumin not only has a strong worm killing effect, but also downregulate the expression of *Sj*HAT in a dose-dependent manner, indicating that *Sj*HAT may be a potential target for curcumin. Notably, DW-3-15 can also inhibit the expression of *Sj*HAT, and has similar antischistosomal efficacy to that of curcumin, which in turn confirming that *Sj*HAT may be a potential target for DW-3-15.

## Conclusion

Our *in vitro* results demonstrated that the natural compound curcumin can potently kill both schistosomula and adult worms of *S*. *japonicum*. Curcumin, which is recognized as a kind of HAT inhibitor, can significantly reduce the mRNA level of *Sj*HAT in a dose-dependent manner. Moreover, DW-3-15, a kind of PZQ derivative, displays schistosomicidal efficacy against juveniles and adults similar to that of curcumin, and can also downregulate *Sj*HAT levels. Both curcumin and DW-3-15 can decrease the expression of the *Sj*HAT gene, enhancing the feasibility of using *Sj*HAT as a potential druggable target. However, further studies are needed to elucidate the characteristics of *Sj*HAT and worm-killing mechanism by downregulating *Sj*HAT.

## Supporting information

S1 Table Downregulation of histone acetyltransferase in female *Schistosoma japonicum* adult worms after treatment with DW-3-15 detected by TMT technique. (DOCX)

S2 Table Raw data of viability scores of male *Schistosoma japonicum* adult worms at 24h, 48h and 72h of incubation of different concentrations of HAT inhibitors, PZQ and DW-3-15. This data was demonstrated in Table 2 and Fig 1.
(XLS)

S3 Table Raw data of viability scores of female *Schistosoma japonicum* adult worms at 24h, 48h and 72h of incubation of different concentrations of HAT inhibitors, PZQ and DW-3-15. This data was demonstrated in Table 3 and Fig 2.
(XLS)

S4 Table Raw data of viability scores of *Schistosoma japonicum* juveniles at 24h, 48h and 72h of incubation of different concentrations of HAT inhibitors, PZQ and DW-3-15. This data was demonstrated in Table 4 and Fig 3.
(XLS)

S1 Fig *Sj*HAT protein levels at 72h of *in vitro* treatment with100 μM DW-3-15 in female *Schistosoma japonicum* adult worms. The control group was incubated with complete DMEM with 0.1% DMSO. The concentration of curcumin, DW-3-15 and PZQ was 100 μM. Student’s *t*-test was applied, with **P<0.01. Western blot data are representative of three independent experiments.
(DOCX)
