# Supplementary material for: Schistosomicidal effects of histone acetyltransferase inhibitors against Schistosoma japonicum juveniles and adult worms in vitro
Source: PLoS Negl Trop Dis. 2024 Aug 19;18(8):e0012428. doi: 10.1371/journal.pntd.0012428 (PMC11361729; doi:10.1371/journal.pntd.0012428)
Supplement: S1 Table — (DOCX) [file pntd.0012428.s001.docx]

S1_Table Downregulation of histone acetyltransferase in female *Schistosoma japonicum* adult worms after treatment with DW-3-15 detected by TMT technique

| Protein accession | Protein description | Gene name | Regulated Type | MW [kDa] | females in DW-3-15 group/females in control group | *P* value | males in DW-3-15 group/males in control group | *P* value |
| --- | --- | --- | --- | --- | --- | --- | --- | --- |
| C1L621 | Histone acetyltransferase OS=Schistosoma japonicum OX=6182 | / | Down | 50.599 | 0.488 | 0.016 | 1.377 | 0.177 |
