# Supplementary material for: Schistosomicidal effects of histone acetyltransferase inhibitors against Schistosoma japonicum juveniles and adult worms in vitro
Source: PLoS Negl Trop Dis. 2024 Aug 19;18(8):e0012428. doi: 10.1371/journal.pntd.0012428 (PMC11361729; doi:10.1371/journal.pntd.0012428)
Supplement: S1 Fig — The control group was incubated with complete DMEM with 0.1% DMSO. The concentration of curcumin, DW-3-15 and PZQ was 100 μM. Student’s t-test was applied, with **P<0.01. Western blot data are representative of three independent experiments. (DOCX) [file pntd.0012428.s005.docx]

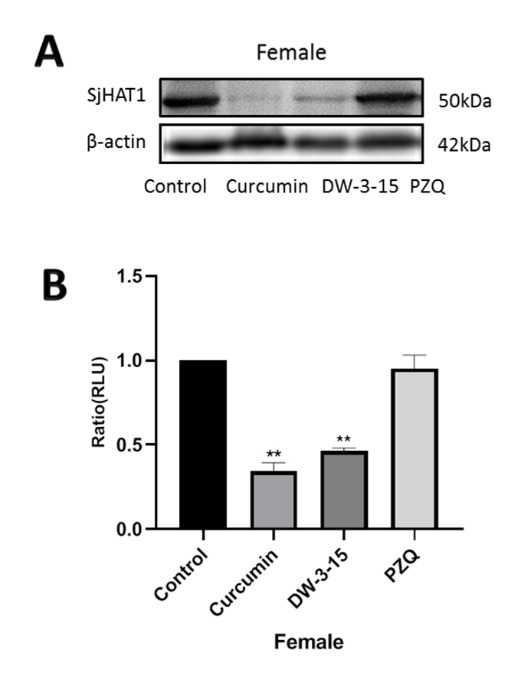


S1_Fig *Sj*HAT protein levels at 72h of in vitro treatment with100 μM DW-3-15 in female *Schistosoma japonicum* adult worms. The control group was incubated with complete DMEM with 0.1% DMSO. The concentration of curcumin, DW-3-15 and PZQ was 100 μM. Student’s *t*-test was applied, with ***P*<0.01. Western blot data are representative of three independent experiments.
